# Characterisation of a Novel A-Superfamily Conotoxin

**DOI:** 10.3390/biomedicines8050128

**Published:** 2020-05-20

**Authors:** David T. Wilson, Paramjit S. Bansal, David A. Carter, Irina Vetter, Annette Nicke, Sébastien Dutertre, Norelle L. Daly

**Affiliations:** 1 Centre for Molecular Therapeutics, Australian Institute of Tropical Health and Medicine, James Cook University, Smithfield, QLD 4878, Australia; david.wilson4@jcu.edu.au (D.T.W.); paramjit.bansal@jcu.edu.au (P.S.B.); 2 Centre for Pain Research, Institute for Molecular Bioscience, The University of Queensland, St Lucia, QLD 4072, Australia; d.carter@imb.uq.edu.au (D.A.C.); i.vetter@imb.uq.edu.au (I.V.); 3 School of Pharmacy, The University of Queensland, Woolloongabba, QLD 4102, Australia; 4 Walther Straub Institute of Pharmacology and Toxicology, Faculty of Medicine, LMU Munich, Nußbaumstraße 26, 80336 Munich, Germany; annette.nicke@lrz.uni-muenchen.de; 5 Institut des Biomolécules Max Mousseron, UMR 5247, Université de Montpellier, CNRS, 34095 Montpellier, France; sebastien.dutertre@umontpellier.fr

**Keywords:** conopeptide, NMR spectroscopy, disulfide framework

## Abstract

Conopeptides belonging to the A-superfamily from the venomous molluscs, *Conus*, are typically α-conotoxins. The α-conotoxins are of interest as therapeutic leads and pharmacological tools due to their selectivity and potency at nicotinic acetylcholine receptor (nAChR) subtypes. Structurally, the α-conotoxins have a consensus fold containing two conserved disulfide bonds that define the two-loop framework and brace a helical region. Here we report on a novel α-conotoxin Pl168, identified from the transcriptome of *Conus planorbis*, which has an unusual 4/8 loop framework. Unexpectedly, NMR determination of its three-dimensional structure reveals a new structural type of A-superfamily conotoxins with a different disulfide-stabilized fold, despite containing the conserved cysteine framework and disulfide connectivity of classical α-conotoxins. The peptide did not demonstrate activity on a range of nAChRs, or Ca^2+^ and Na^+^ channels suggesting that it might represent a new pharmacological class of conotoxins.

## 1. Introduction

Cone snail venoms comprise mainly small peptides, termed conotoxins, and represent one of the most extensive libraries of bioactive compounds from marine creatures. Conotoxins generally have selectivity and potency for a range of ion channels and G-protein coupled receptors and consequently have been useful as pharmacological tools and therapeutic leads [1]. Several conotoxins have been tested in clinical trials, with an N-type calcium channel blocker from *Conus magus* (ω-MVIIA) approved by the Federal Drug Administration (FDA) as Prialt^®^ for the treatment of chronic pain [2,3,4]. While the majority of studies aimed at developing conotoxins as drug leads focussed on treatment of pain (e.g., ω-CVID and χ-MrIA [5]) other studies have expanded the potential applications of conotoxins. Conantokin G, a *N*-methyl-D-aspartate (NMDA) antagonist from *Conus geographus*, has been of interest for development as an anticonvulsant [6], while more recent studies have shown antimycobacterial activity (O1_cal29b from *Californiconus californicus*) [7] and inhibitory effects against the growth of lung cancer cells (TxID from *Conus textile*) [8]. The venom of a single species can contain hundreds of different peptides and with at least 750 different species of cone snails [9,10], it is estimated that more than 1 million unique peptides exist [10]. However, despite extensive study in the field, we have currently sampled less than 1% of this diversity [10].

Conotoxins have been classified into various gene superfamilies based on signal sequence conservation, in addition to classification into families based on cysteine framework and receptor targets [11]. The number of disulfide bonds is typically 2-4, but the connectivity can vary even amongst conotoxins containing the same number of cysteine residues, as can the bioactivity. The known cone snail venom peptide sequences and their known functions have been collated in the database Conoserver [12,13]. Twenty-nine superfamilies and thirty different cysteine frameworks have been identified in Conoserver to date, highlighting the diversity in the sequences of conotoxins. The A-superfamily is one of the most well characterised, with the majority containing cysteine framework I (CC-C-C). This framework is primarily associated with the α-conotoxin family, members of which specifically antagonise the nicotinic acetylcholine receptors (nAChRs) [14,15]. nAChRs are ligand-gated ion channels involved in a range of physiological and pathophysiological processes, including muscle contraction, pain sensation and nicotine addiction. They are classified as muscle-type and neuronal, and have been implicated in neurological disorders such as Parkinson’s and Alzheimer’s diseases [16] making them potential drug targets. nAChRs exist as homopentamers or heteropentamers comprising a range of different subunits [17]. α-Conotoxins are one of the most medically relevant families of conotoxins, highlighted by a cyclic version of Vc1.1 displaying oral activity in an animal model of neuropathic pain [3,18]. Additional engineering studies have further highlighted the potential of this peptide in drug design [19,20].

**Figure 1:**
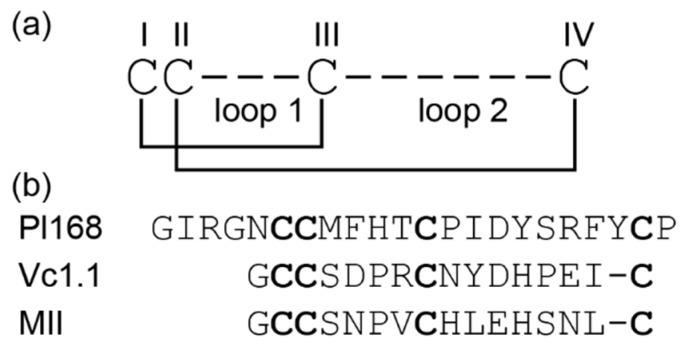
Framework and sequences of α-conotoxins. (**a**) Framework I is represented highlighting the two inter-cysteine loops; the number of residues within each of these loops is used to define the α-conotoxin class. In addition, the globular disulfide bond connectivity present in α-conotoxins is also shown. (**b**) The sequence of Pl168 from *Conus planorbis*, which contains a 4/8 framework. The sequences of related 4/7 α-conotoxins, Vc1.1 and MII are also shown. Cysteine residues are highlighted in bold.

α-Conotoxins are generally less than 20 residues in length, have a CysI-CysIII, CysII-CysIV disulfide connectivity and the majority have a 4/7 loop spacing, which represents 4 residues in the first inter-cysteine loop and 7 in the second loop (Figure 1a). A well-studied example containing this loop spacing is Vc1.1 [21]. Several other loop spacings have been identified in the α-conotoxin family, and the size of the loops correlates to some extent with specificity for different nAChR subtypes. For example, 3/4 α-conotoxins target homomeric neuronal nAChRs, 3/5 α-conotoxins target muscle-type nAChRs and 4/4, 4/6 and 4/7 α-conotoxins target different heteromeric and/or homomeric neuronal nAChRs [14].

The structures of several α-conotoxins have been determined using nuclear magnetic resonance (NMR) spectroscopy. This technique is well suited to the determination of the structures of α-conotoxins because of their small size, high aqueous solubility and relatively well-defined structures [22]. In addition, some studies used X-ray crystallography to study α-conotoxins either in isolation or in complex with binding partners. Despite the variation in inter-cysteine loop sizes across the family, the majority of α-conotoxins are characterised by a small helical structure, which is braced by the CysI-CysIII disulfide bond. The additional disulfide bond connecting CysII-CysIV generally tethers the C-terminal region to the N-terminus.

Here we show that a minor change in the inter-cysteine loop spacing in framework I can have a significant impact on the structure and bioactivity. Pl168, a 22-residue framework I peptide that contains an unusual 4/8 spacing, was identified in the transcriptome of *Conus planorbis* as a new α-conotoxin [23]. The sequence of Pl168, along with the sequences of two well characterised α-conotoxins, Vc1.1 and MII, are given in Figure 1b. The three-dimensional structure of Pl168 differs significantly from the characteristic α-conotoxin fold and the peptide does not block a range of nAChRs or Ca^2+^ and Na^+^ channels, indicating that it might represent a new pharmacological class of A-superfamily toxin.

## 2. Experimental Section

### 2.1. Peptide Synthesis, Purification and Characterisation

Synthetic Pl168 was manually synthesised using standard solid-phase peptide synthesis fluorenylmethyloxycarbonyl (Fmoc) methods and 2-chlorotrityl-chloride resin. The Fmoc protected amino acids (Auspep, Australia) were activated using *O*-(1*H*-6-Chlorobenzotriazole-1-yl)-1,1,3,3-tetramethyluronium hexafluorophosphate (HCTU) (Iris, Germany) and coupled on resin with *N*,*N*-diisopropylethylamine (DIPEA) (Auspep, Australia)/dimethylformamide (DMF) (Auspep, Australia) by stepwise solid-phase peptide synthesis chemistry. Cleavage of the peptide chain from the solid support was achieved with a mixture of trifluoroacetic acid (TFA) (Auspep, Australia):triisopropylsilane (TIPS) (Auspep, Australia):H_2_O (95%:2.5%:2.5% *v*/*v*) followed by purging with nitrogen to evaporate TFA. The peptide was then precipitated in ice-cold diethyl ether (Auspep, Australia) and dissolved in 50% acetonitrile (Sigma, Australia):50% H_2_O:0.1% TFA (*v*/*v*) and subsequently lyophilised to dryness. Crude peptide was purified by reversed-phase high performance liquid chromatography (RP-HPLC) on a Phenomenex Jupiter C_18_ preparative column (300 Å, 10 μm, 250 × 21.2 mm) (Phenomenex, Torrance, CA, USA), using a gradient of 0-60% solvent Β (Solvent A: 99.95% H_2_O:0.05% TFA; Solvent Β: 90% acetonitrile:10% H_2_O:0.045%TFA) over 60 min. Collected fractions were analysed using a SCIEX 5800 matrix-assisted laser desorption ionisation (MALDI) time-of-flight (TOF)/TOF mass spectrometer (SCIEX, Foster City, CA, USA) and then lyophilised. Formation of the disulfide bonds was carried out in ammonium bicarbonate pH 8.0 (Sigma, Australia) at room temperature and the major isomer from the oxidation reaction purified using RP-HPLC and the mass confirmed using MALDI-TOF mass spectrometry (SCIEX, Foster City, CA, USA).

### 2.2. NMR Spectroscopy

Lyophilised peptide was dissolved in 90% H_2_O:10% D_2_O at a concentration of approximately 0.2 mM. All NMR spectra were acquired on a Bruker 600 MHz AVANCE III NMR spectrometer (Bruker, Karlsruhe, Germany) equipped with a cryogenically cooled probe. Two-dimensional ^1^H-^1^H TOCSY, ^1^H-^1^H NOESY, ^1^H-^1^H DQF-COSY, and collected at 290 K were used for sequence-specific assignments and structure calculations. Thus, ^1^H-^15^N HSQC, and ^1^H-^13^C HSQC spectra were acquired for carbon and nitrogen chemical shifts, respectively. All spectra were recorded with a 1 s interscan delay using standard Bruker pulse sequences with an excitation sculpting scheme for solvent suppression. Two-dimensional spectra were collected over 4096 data points in the f2 dimension and 512 increments in the f1 dimension over a spectral width of 12 ppm. Homonuclear NOESY and TOCSY spectra were acquired with a mixing time of 200 and 250 ms, and a spin lock time of 80 ms, respectively. All spectra were processed using Bruker TopSpin (Version 3.5pl7) and assigned using CCPNMR analysis [24] based on the approach described in Wüthrich et al. [25]. The αH secondary shifts were determined by subtracting random coil ^1^H NMR chemical shifts from the experimental αH chemical shifts [26].

### 2.3. Structure Calculations

Structures were calculated with the CYANA program using an automated NOE assignment protocol [27]. Torsion-angle restraints were predicted using TALOS-N [28] and hydrogen bonds predicted based on preliminary structures calculated without disulfide bond restraints. Calculations were also performed with the three possible disulfide bond connectivities to determine the most likely connectivity. A set of 100 final structures was calculated with the globular disulfide connectivity and 20 structures with the lowest target function chosen to present the final ensemble. Structures were visualized and the root-mean-square deviation (RMSD) values were assessed using MOLMOL [29].

### 2.4. Electrophysiological Measurements

Rat nAChR cDNAs were provided by J. Patrick, Baylor College of Medicine, Houston, TX, and subcloned into the oocyte expression vector pNKS2. The cRNA was synthesized with the SP6 mMessage mMachine kit (Ambion, Austin, TX, USA), and *Xenopus laevis* (Nasco International) oocytes were kindly provided by Prof. Luis Pardo, Göttingen). Oocytes were injected with 50 nL aliquots of cRNA (0.05 mg/mL). The nAChR subunits of heteromeric receptors were mixed at the ratio of 1:1 (α3:β2) or 5:1 (α4:β2).

Recordings were performed as described [30] in ND96 (96 mM NaCl, 2 mM KCl, 1 mM CaCl_2_, 1 mM MgCl_2_, and 5 mM 4-(2-hydroxyethyl)-1-piperazineethanesulfonic acid (HEPES) at pH 7.4). Briefly, current responses to 100 μM acetylcholine (ACh) or 100 μM nicotine (in the case of α7) were recorded at −70 mV using a Turbo Tec 05X Amplifier (NPI Electronic, Tamm, Germany) and Cell Works software. A fast and reproducible solution exchange (<300 ms) was achieved with a 50 μL funnel-shaped oocyte chamber combined with a fast solution flow (∼150 μL s^−1^) fed through a custom-made manifold mounted immediately above the oocyte. Agonist pulses were applied for 2 s at 4 min intervals. Peptide up to 100 μM was applied for 3 min in a static bath.

### 2.5. FLIPR^Tetra^ Ion Channel Assays

The effect of Pl168 on human ion channels was assessed using a high-throughput Ca^2+^ imaging assay as previously described [31,32,33,34]. In brief, SH-SY5Y human neuroblastoma cells (ATCC) were cultured in Roswell Park Memorial Institute (RPMI) medium supplemented with L-glutamine (1 mM) and 15% foetal bovine serum and maintained at 37 °C/5% CO_2_. Cells were plated on black-walled 384-well imaging plates (Corning, NY, USA) 48 h prior to loading with Calcium 4 No-wash dye (Molecular Devices, Sunnyvale, CA) in physiological salt solution (PSS, composition in mM: 140 NaCl, 11.5 glucose, 5.9 KCl, 1.4 MgCl_2_, 1.2 NaH_2_PO_4_, 5 NaHCO_3_, 1.8 CaCl_2_, 10 HEPES, pH 7.4). Fluorescence responses (excitation 470–495 nm; emission 515–575 nm) were measured at 1 s intervals using a FLIPR^Tetra^ fluorescence imaging plate reader (Molecular Devices, Sunnyvale, CA), with peptide (30 µM) added 300 s prior to stimulation of ion channel specific responses, followed by a further 300 reads. Ca_V_2.2 responses were elicited by addition of KCl (90 mM)/CaCl_2_ (5 mM) in the presence of nifedipine (10 µM); α7 nAchR responses by choline (30 µM) in the presence of PNU-120596 (10 µM); α3-containing nAChR responses by nicotine (30 µM); Ca_V_1 responses by KCl (90 mM)/CaCl_2_ (5 mM) in the presence of ω-conotoxin CVIF (10 µM); and Na_V_ responses by veratridine (50 µM). Data was analysed using ScreenWorks 3.2.0.14 (Molecular Devices, Sunnyvale, CA, USA) and expressed as response over baseline, with baseline defined as 10 reads prior to agonist addition.

## 3. Results

### 3.1. Peptide Synthesis and Characterisation

To allow structural and functional characterisation of Pl168, the peptide was synthesised using Fmoc chemistry and oxidation of the cysteine residues to form disulfide bonds was carried out in ammonium bicarbonate pH 8.0 at room temperature. A major isomer was present in the oxidation reaction and was purified using RP-HPLC and the mass analysed using MALDI-TOF mass spectrometry. The sample was lyophilised and stored at 4 °C until subsequent analyses were carried out.

### 3.2. Structural Characterisation

The three-dimensional structure of Pl168 was determined using NMR spectroscopy. The spectra display sharp peaks, and only one conformation is evident based on the number of amide proton peaks. This qualitative analysis suggests that the two proline residues in the sequence are not in cis/trans isomerisation, a phenomenon which is relatively common in small peptides. Backbone and side-chain assignments were determined using established procedures [25] and dihedral angle restraints were predicted based on the chemical shift assignments using TALOS [28]. Slowly exchanging amide protons were analysed by dissolving lyophilized peptide in 100% D_2_O and recording one-dimensional and TOCSY spectra over time. More than eight amide protons were evident in the spectra following 10 min of dissolution in D_2_O, with three (Thr11, Tyr20 and Cys21) still present after three hours. The protection of amide protons from the solvent indicates they are involved in hydrogen bonds. Preliminary structures were calculated using CYANA, based on the predicted dihedral angle restraints and incorporating a protocol for automated assignment of the NOESY inter-residue cross-peaks to derive distance restraints. Analysis of the preliminary structures and slowly exchanging amide protons allowed the incorporation of hydrogen bond restraints into the calculations.

**Figure 2:**
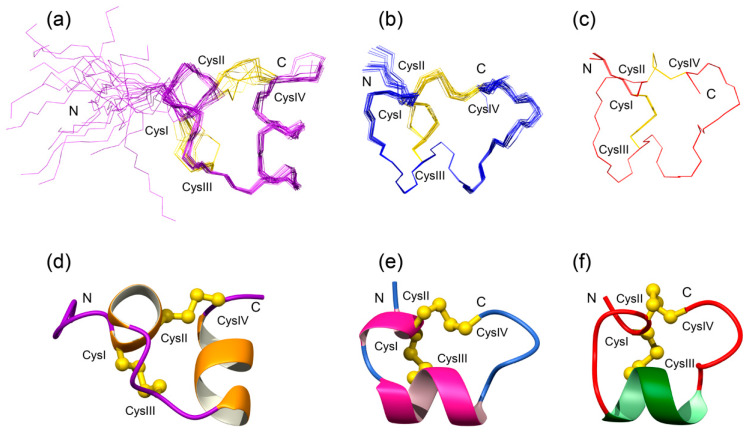
Three-dimensional structures of Pl168 (**a**,**d**), Vc1.1 (**b**,**e**; PDB code 2h8s) and MII (**c**,**f**; PDB code 1mii). Superposition of the lowest energy structures are shown at the top of the diagram and the ribbon representation showing the secondary structure shown at the bottom. The disulfide bonds are shown as yellow lines on the top of the figure, and in ball-and-stick format on the bottom.

Initial structures were calculated without disulfide bond restraints. Analysis of these structures indicated that Cys6 forms a disulfide bond with Cys12, based on 11 out of 15 structures having sulfur-sulfur distances for these two cysteine residues in close proximity. The sulfur atoms of Cys7 and Cys21 were not in close proximity to other cysteine residues. The presence of the Cys6-Cys12 disulfide bond implies that the peptide contains the globular disulfide connectivity (CysI-CysIII, CysII-CysIV) present in α-conotoxins. To confirm this is the likely connectivity, structures were calculated with the three different disulfide connectivities (Table 1). Consistent with the structures calculated without disulfide bond restraints, the globular disulfide bond connectivity had the lowest target function of all three connectivities. An overlay of the 20 lowest energy structures incorporating the globular connectivity, and the secondary structure present in Pl168 is given in Figure 2. The main elements of secondary structure are α-helices from Cys6 to Phe9, and Ile14 to Tyr20. The N-terminal region, prior to the first cysteine residue, is disordered in the structures, consistent with the lack of NOEs in this region. By contrast, residues 6–21 are well defined. The structural statistics for the final ensemble of structures are given in Table 2.

### 3.3. Electrophysiology

The influence of Pl168 on a range of cloned nAChR subtypes expressed in *Xenopus laevis* oocytes was assessed by two-electrode voltage clamp measurements as previously described [30]. No effect was observed on the α7, α4β2, α3β2 or muscle-type nAChRs at peptide concentrations up to 100 μM.

### 3.4. FLIPR^Tetra^ Ion Channel Assays

**Figure 3:**
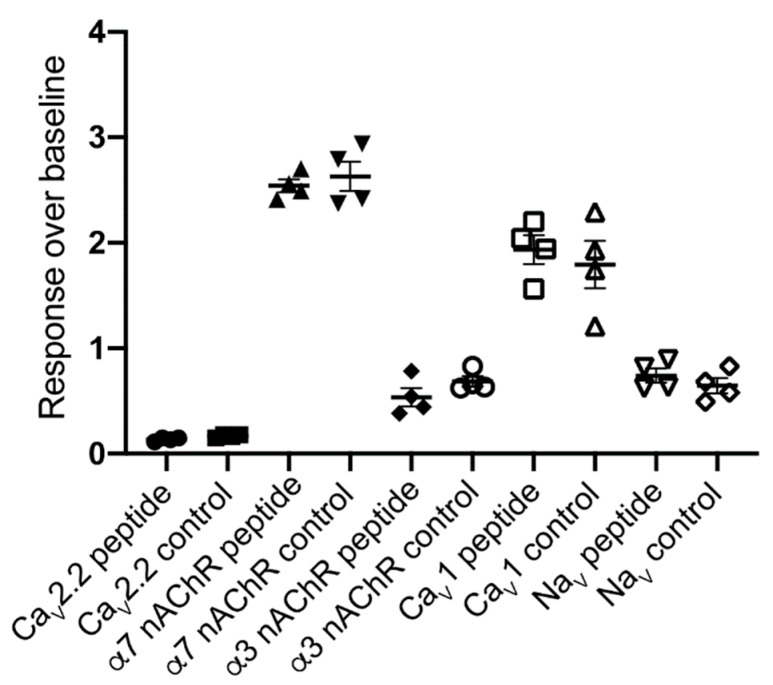
The effect of Pl168 (30 µM) on ion channel responses assessed using fluorescent Ca^2+^ imaging in the human neuroblastoma cell line SH-SY5Y endogenously expressing nAChR, Ca_V_ and Na_V_ channels. No effect was observed with the exception of a small (18%) inhibition of N-type (Ca_V_2.2) voltage-gated Ca^2+^ channel responses. (●) Pl168 (30 µM) on Ca_V_2.2 channels; (■) Control KCl (90 mM)/CaCl_2_ (5 mM) in the presence of nifedipine (10 µM) on Ca_V_2.2 channels; (▲) Pl168 (30 µM) on α7 nAchRs; (▼) Control α7 nAchR response by choline (30 µM) in the presence of PNU-120596 (10 µM); (◆) Pl168 (30 µM) on α3 nAchRs; (⭘) Control α3-containing nAChR response by nicotine (30 µM); (☐) Pl168 (30 µM) on Ca_V_1 channels; (△) Control Ca_V_1 response by KCl (90 mM)/CaCl_2_ (5 mM) in the presence of ω-conotoxin CVIF (10 µM); (▽) Pl168 (30 µM) on Na_V_ channels; (◇) Control Na_V_ channel response by veratridine (50 µM).

The effect of Pl168 (30 µM) on ion channel responses (response over baseline; mean ± S.E.M, *n* = 4) was assessed using fluorescent Ca^2+^ imaging in the human neuroblastoma cell line SH-SY5Y endogenously expressing nAchR, Ca_V_ and Na_V_ channels. Pl168 (30 µM) had no effect on α7 nAChR responses (control, 2.6 ± 0.14; Pl168, 2.5 ± 0.06), no effect on nicotine-induced α3 nAChR responses (control, 0.69 ± 0.05; Pl168, 0.54 ± 0.09), no effect on L-type (Ca_V_1) voltage-gated Ca^2+^ channel responses (control, 1.8 ± 0.23; Pl168, 1.9 ± 0.14) or voltage-gated Na^+^ channel responses (control, 0.65 ± 0.07; Pl168, 0.74 ± 0.07), and only a small (18%) inhibition of N-type (Ca_V_2.2) voltage-gated Ca^2+^ channel responses (control, 0.17 ± 0.008; Pl168, 0.14 ± 0.01) (Figure 3). Addition of the peptide also caused no increase in Ca^2+^, suggesting it does not act as an agonist at endogenously expressed ion channels or receptors linked to Ca^2+^ signalling.

## 4. Discussion

We have determined the three-dimensional structure of an A-superfamily conotoxin containing an unusual 4/8 spacing and shown that it represents a new structural sub-family. α-Conotoxins, the major family of A-superfamily peptides, have been well studied with more than 70 structures submitted to the Protein Data Bank. In general, the fold is very similar across the family, but we show here that an additional residue in loop 2 appears to have a significant effect on the structural fold.

Pl168 was synthesized without selective protection of the cysteine residues, but nonetheless a major isomer was produced, which was purified and structurally analysed using NMR spectroscopy. Structural analysis indicated that the globular disulfide bond connectivity was present in the synthetic version of Pl168. The Pl168 sequence was identified from transcriptomic data and therefore a co-elution with the native material was not possible. Although unlikely, it is conceivable that the native peptide present in the venom displays a different connectivity given that the folding conditions can have a significant influence on the isomers present. For instance, a recent study showed that recombinant expression of the α-conotoxin TxIA resulted in the ribbon isomer in contrast to synthetic studies which allowed production of the globular isomer [35]. In the absence of a direct comparison of the synthetic peptide with native material we cannot say definitively that this connectivity is present in the venom, but the well-defined structure displayed by the synthetic version of Pl168 and the propensity of A-superfamily framework I toxins to contain the globular connectivity is consistent with the synthetic peptide being equivalent to the native peptide.

The structures of Pl168 comprise two helical regions connected by a loop region. Comparison of Pl168 with the 4/7 conotoxin Vc1.1 indicates that the folds are similar in that both are characterised by α-helices braced by two disulfide bonds as shown in Figure 2. It should be noted, however, that not all the Vc1.1 structures in the ensemble [36] contain the N-terminal helix, and many α-conotoxins only contain the one helical region centered around CysIII as shown for α-conotoxin MII [37] (Figure 2). However, the structural differences between α-conotoxins such as Vc1.1 and MII, and Pl168 relate to the bracing of the disulfide bonds. Whereas CysIII in Vc1.1 and MII is located within the α-helix, Cys III in Pl168 is present in the loop region and this distinction prevents an effective superposition of these structures. These structural differences, and the presence of a related peptide in *Conus planorbis* with only one residue different to Pl168 (Y20N mutation; GenBank: ADJ67509.1), suggests that Pl168 could represent a new structural class and possibly a new sub-family of conotoxins.

Extensive mutational studies have been done on a range of α-conotoxins, which have identified residues important for bioactivity, including a recent study on MilIA, the first conotoxin isolated from *Conus milneedwardsi* [38]. Interestingly, mutations in MilIA allowed elucidation of residues important in switching between muscle and neuronal nAChRs preference. However, the α-conotoxin family is large with diverse inter-cysteine loop sequences and there does not appear to be a common pharmacophore. The lack of activity of Pl168 against a range of nAChRs suggests that the overall three-dimensional structural and side-chain orientations are not optimal for interaction with the mammalian nAChRs tested in this study. However, given the range of different nAChRs subunits, it is still possible that it inhibits a subtype not tested or a prey-specific nAChR (*C. planorbis* feeds on polychete worms). Although Pl168 contains a conserved signal sequence with other α-conotoxins, it might target another receptor or ion channel as peptides from the A-superfamily are very diverse in their sequence and bioactivity [11]. However, the lack or minimal activity against Ca^+^ and Na^+^ channels suggests that a wide screen is required to determine the bioactivity of this peptide.

## Figures and Tables

**Table 1:** Analysis of Pl168 structures calculated with the three possible disulfide bond connectivities.

Connectivity	Fold	Target Function ^1^
Cys6-Cys12, Cys7-Cys21	Globular	0.046 ± 0.037
Cys6-Cys21, Cys7-Cys12	Ribbon	1.23 ± 0.099
Cys6-Cys7, Cys12-Cys21	Beads	3.3 ± 0.090

^1^ Average target function from 15 structures calculated using CYANA.

**Table 2:** Structural statistics for pl168 with a globular disulfide connectivity.

Experimental Restraints	
Interproton distance restraints	
*Intraresidue*	57
*Sequential*	50
*Medium range (i–j < 5)*	17
*Long range (i–j ≥ 5)*	8
*Total*	132
Dihedral-angle restraints	30
Hydrogen bonds (2 per bond)	12
**R.m.s. deviations from mean coordinate structure (Å) (in residues 6–21)**	
Backbone atoms	0.44 +/− 0.14
All heavy atoms	1.51 +/− 0.25
Ramachandran Statistics *	
% in most favoured region	77.8
% in additionally allowed region	22.2

* Based on the PROCHECK analysis https://servicesn.mbi.ucla.edu/PROCHECK/.
